# Effects of the Mediterranean Diet before and after Weight Loss on Eating Behavioral Traits in Men with Metabolic Syndrome

**DOI:** 10.3390/nu9030305

**Published:** 2017-03-19

**Authors:** Élise Carbonneau, Marie-Michelle Royer, Caroline Richard, Patrick Couture, Sophie Desroches, Simone Lemieux, Benoît Lamarche

**Affiliations:** 1 Institute of Nutrition and Functional Foods, Laval University, 2440 Hochelaga Boulevard, Quebec, QC G1V 0A6, Canada; elise.carbonneau.1@ulaval.ca (E.C.); mmroyer@hotmail.com (M.-M.R.); 2 Department of Agricultural Food and Nutritional Science, University of Alberta, 410 Agriculture/Forestry Centre, Edmonton, AL T6G 2P5, Canada; cr5@ualberta.ca; 3 Lipid Research Center, CHU de Quebec, 2705, Laurier Boulevard, Quebec, QC G1V 4G2, Canada; patrick.couture@crchul.ulaval.ca; 4 Institute of Nutrition and Functional Foods, Laval University, 2440 Hochelaga Boulevard, Quebec, QC G1V 0A6, Canada; sophie.desroches@fsaa.ulaval.ca (S.D.); simone.lemieux@fsaa.ulaval.ca (S.L.)

**Keywords:** Mediterranean diet, metabolic syndrome, weight loss, Three-Factor Eating Questionnaire, cognitive restraint, disinhibition, susceptibility to hunger

## Abstract

The objective of this study was to investigate the impact of the Mediterranean diet (MedDiet) consumed before and after weight loss on eating behavioral traits as measured by the Three-Factor Eating Questionnaire (TFEQ) in men with metabolic syndrome (MetS). In this fixed sequence study, 19 men with MetS (National Cholesterol Education Program-Adult Treatment Panel III (NCEP-ATPIII) criteria), aged between 24 and 62 years, first consumed a five-week standardized North American control diet followed by a five-week MedDiet, both under weight-maintaining controlled-feeding conditions. This was followed by a 20-week caloric restriction weight loss period in free-living conditions, without specific recommendations towards adhering to the principles of the MedDiet. Participants were finally subjected to a final five-week MedDiet phase under isoenergetic controlled-feeding conditions. The MedDiet before weight loss had no impact on eating behavioral traits. Body weight reduction by caloric restriction (−10.2% of initial weight) was associated with increased cognitive restraint (*p* < 0.0001) and with reduced disinhibition (*p* = 0.02) and susceptibility to hunger (*p* = 0.01). Feeding the MedDiet for five weeks under isoenergetic conditions after the weight loss phase had no further impact on eating behavioral traits. Results of this controlled-feeding study suggest that consumption of the MedDiet per se has no effect on eating behavioral traits as measured by TFEQ, unless it is combined with significant weight loss.

## 1. Introduction

The protective role of the Mediterranean diet (MedDiet) in the realm of cardiovascular disease prevention has been investigated extensively in the last decade [1,2,3,4,5]. The MedDiet has been claimed to protect against the development of metabolic syndrome (MetS) not only because of its beneficial role on cardiovascular risk factors [6,7], but also due to possible effects on the regulation of body weight [8,9,10,11,12]. The satiating property of the MedDiet, which is partly attributed to its high nutrient density combined with its low energy density, is a key factor responsible for its effect on body weight [13]. In fact, it has been suggested that consumption of satiating, low energy density diets, such as the MedDiet, reduces spontaneous energy intake, which in turn favors weight loss [14]. This reduction in energy intake is attributed to the high satiety quotient of low energy density foods, which reflects a higher satiating effect of a given amount of energy for low versus high energy density food [15,16,17]. Studies have suggested that eating less than wanted is associated with increased cognitive restraint [18], a construct that defines the intent to restrict food intake in order to control body weight. Other eating behavioral traits include disinhibition and susceptibility to hunger [18]. Disinhibition refers to overconsumption of food in response to various stimuli associated with losing control of food intake [18]. Susceptibility to hunger refers to one’s food intake in response to one’s feelings and perceptions of hunger [18]. We have hypothesized that eating more than wanted in a context of isoenergetic consumption of the MedDiet reduces cognitive restraint and susceptibility to hunger, while being neutral on disinhibition [19,20].

To the best of our knowledge, very few studies have documented the effect of the MedDiet on these eating behavioral traits. This is of particular importance considering the impact of the MedDiet on weight control. Furthermore, the majority of studies on eating behaviors and weight loss have been conducted in women. Therefore, the aim of this study was to evaluate the impact of the MedDiet, with and without weight loss, on eating behavioral traits as determined by the Three-Factor Eating Questionnaire [18] in men with MetS.

## 2. Materials and Methods

### 2.1. Subjects

At baseline, the study sample included 29 men from the Quebec metropolitan area, 18 to 65 years of age, and affected by the MetS, as defined by the NCEP-ATPIII criteria [21]. Exclusion criteria were smoking, self-reported history of cardiovascular diseases or type 2 diabetes, and use of prescribed lipid lowering or hypertension medication. All participants signed an informed consent document approved by the Ethics committee of Laval University before entering the study. The study was conducted in accordance with the Declaration of Helsinki and was registered at ClinicalTrials.gov (NCT00988650).

### 2.2. Study Design

**Figure 1:**
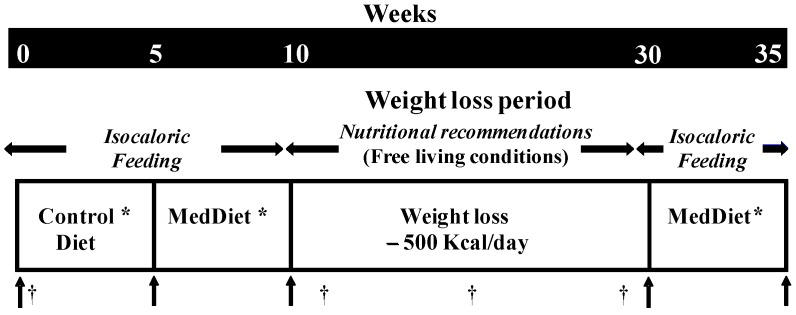
Study design. * Controlled feeding phases of the study (daily weighting and adjustment of energy intake). † Food and physical activity records (three days). **↑** Anthropometric and eating behavior measurements. MedDiet: Mediterranean Diet.

This fixed sequence study comprised three five-week isoenergetic feeding phases as well as a 20-week weight loss (WL) period (Figure 1) as detailed previously [21]. Subjects first consumed a North American control diet for five weeks under isoenergetic weight-maintaining conditions. This was followed by the MedDiet also consumed under isoenergetic weight-maintaining conditions for a five-week period. These two phases aimed at examining the impact of the MedDiet on eating behavioral traits in the absence of WL. After the first two isoenergetic phases of the study, participants were subjected to a 20-week calorie restriction WL program in free-living conditions. No food was provided and subjects were specifically instructed not to adhere to the principles of the MedDiet during the WL phase. The focus was mainly on restricting caloric intake to achieve a 500 kcal deficit daily. This was done mainly by proposing reductions in serving sizes and by substituting fiber rich foods for energy dense foods such as soft drinks, sweets, fast food and other high fat/sugary foods. Nutritional guidance was provided during that period through individual bi-weekly sessions with a dietitian as well as support over the phone. Only participants who lost a minimum of 5% of their initial body weight were eligible for the final part of the study, which consisted of a final five-week isoenergetic, weight-stabilizing and controlled feeding phase on the MedDiet.

During the three isoenergetic phases, all foods and beverages were provided to the participants at the Clinical Investigation Unit of the Institute of Nutrition and Functional Foods (Quebec, Canada). Participants were instructed to consume only the meals provided and to maintain their usual level of physical activity. Required energy intake for the isoenergetic phases of the study was estimated using a three-day food record completed by eligible participants at screening. Fluctuations in body weight were measured daily and energy intake was adjusted accordingly to ensure weight-maintaining conditions. Compliance to the diets was measured using a checklist provided to participants, which identified any non-prescribed food eaten or prescribed food that had not been entirely consumed. The composition of the control diet and the MedDiet used in this study is presented in Table 1.

### 2.3. Anthropometry Measurements

Weight (BWB-800 Digital scale, Tanita), height, waist and circumferences (to the nearest cm) and body mass index (BMI, weight/height^2^) were measured at baseline and at the end of each phase (see Figure 1) according to standardized methods [22].

### 2.4. Eating Behaviors Measurements

Eating behavioral traits were assessed using a French version of the Three-Factor Eating Questionnaire (TFEQ) at baseline and after each phase of the study (Figure 1). This 51-item self-administrated questionnaire measures three dimensions of eating behaviors: cognitive restraint, disinhibition, and susceptibility to hunger [18] as well as their subscales [23,24]. For all scales and subscales, higher scores reflect more pronounced behavioral traits.

Cognitive restraint (on a scale of 0 to 21 points) has been traditionally divided into rigid control, which is defined as a dichotomous, all-or-nothing approach to eating (e.g., ‘counting calories’), and flexible control, which is considered as a gradual approach to eating (e.g., ‘small helping as a means of weight control’) [23]. Other subscales of cognitive restraint include: strategic dieting behaviors, specifically those used to control weight (e.g., ‘consciously eat less than wanted’); attitude to self-regulation, an assessment of a subject’s overarching perspectives concerning eating and weight control (e.g., ‘life is too short to worry about dieting’ (reverse-coded)); and avoidance of fattening foods, the conscious behavior of limiting this specific category of food (e.g., ‘how likely to shop for low calorie food’) [24].

Disinhibition (on a scale of 0 to 16 points) is considered to be composed of three subscales: habitual susceptibility to disinhibition (circumstantial predispositions to recurrent disinhibition), emotional susceptibility to disinhibition (disinhibition related to negative affective states), and situational susceptibility to disinhibition (disinhibition initiated by specific environmental cues) [24].

Susceptibility to hunger (on a scale of 0 to 14 points) can be divided into two subscales: internal and external hunger. Internal hunger refers to the type of hunger that is interpreted and regulated internally (e.g., ‘I often feel so hungry that I just have to eat something’), whereas external hunger is triggered by external cues (e.g., ‘Being with someone who is eating often makes me hungry enough to eat also’) [24].

### 2.5. Determination of Mediterranean Dietary Score (MedScore)

A MedScore was calculated on the basis of the Mediterranean pyramid as previously described [25]. The score ranges from 0 to 44 points, 44 indicating that the typical MedDiet is fully achieved. The actual composition of the control diet and MedDiet provided to participants during the feeding phases was used to calculate the MedDiet score. The MedScore during the WL phase was calculated from three-day weighed food records (two randomly selected weekdays and one weekend day) collected on three separate occasions during that period (Figure 1).

### 2.6. Statistical Analyses

Data are presented as means and standard deviations unless stated otherwise. Statistical analyses were performed using the SAS software version 9.1 (SAS Institute Inc., Cary, NC, USA). The MIXED procedure for repeated measures was used to compare the impact of each phase on weight and eating behaviors. The Tukey-Kramer adjustment from the MIXED model was used to assess multiple comparisons of the diets when a significant treatment effect was found. Variables that were not normally distributed were log transformed prior to analysis. Differences or associations at *p* < 0.05 were considered significant.

## 3. Results

Of the initial 29 participants who were enrolled in this study, two of them discontinued their participation for personal reasons unrelated to the study protocol. A total of eight subjects were also excluded because of their lack of compliance to the study requirements. Seven of those were excluded because they did not achieve the predetermined 5% WL target to be eligible for the last phase of the study. Consequently, the comparison of the phases before and after WL is based on 19 participants.

The MedDiet compared with the North American control diet included more whole grain products, fruits and vegetables, legumes, nuts, cheese and yogurt, fish, olive oil, and red wine, and less eggs, sweets, and red meat (Table 1). Baseline characteristics of the 19 subjects are shown in Table 2. Men were middle-aged and all had MetS. The mean scores for cognitive restraint, disinhibition and susceptibility to hunger were relatively low at baseline, considering the scales of these behavioral traits. The overall compliance to the diets during the isoenergetic feeding phases of the study, calculated using a food checklist, was 97.9% ± 1.8%. The MedScores during the North American control diet and the MedDiet before and after WL were 20 and 37, respectively (Table 1).

### 3.1. Weight Changes

Variations in body weight of the 19 participants ranged from −2.2 kg to +1.1 kg during the control diet and from −2.8 kg to +1.4 kg during the MedDiet, before WL. Mean changes were not significant. As shown in Table 3, the 20-week caloric restriction WL period led to a mean reduction of 10.2% ± 2.9% in body weight (ranging from 5.0% to 12.8%, *p* < 0.0001). The MedScore during the free-living WL phase was 26 ± 5 points, indicating that participants were not particularly adhering to the principles of the MedDiet to lose weight. There was no further significant change in body weight during the last five-week weight-stabilizing isoenergetic MedDiet.

### 3.2. Eating Behaviors

Changes in the eating behavioral traits are presented in Table 3. The North American control diet had no impact on any of the eating behavioral traits measured by the TFEQ compared with the baseline values (shown in Table 2). Consumption of the MedDiet under weight-maintaining conditions before WL also had no significant effect on eating behavioral traits compared with the control diet (Table 3). As expected, WL in free-living conditions induced significant changes in several behavioral traits. For example, the free-living WL phase led to a significant increase in cognitive restraint, including rigid control, and attitude to self-regulation compared with the control and MedDiet before weight loss (*ps* < 0.05). WL also led to significant reductions in total disinhibition and in situational disinhibition compared with the pre-WL diets (*ps* < 0.05). Finally, consumption of the MedDiet after WL was associated with a significantly higher cognitive restraint scores and with lower disinhibition and susceptibility to hunger scores compared with the consumption of the MedDiet in the absence of WL (*ps* < 0.05).

## 4. Discussion

To the best of our knowledge, this is the first study to investigate how consumption of a MedDiet, with and without significant weight loss, modifies eating behavioral traits measured by the TFEQ. Contrary to our hypothesis, our results suggested that the MedDiet under isoenergetic conditions has no impact on eating behavioral traits in men with MetS. Indeed, behavior changes observed in the present study were related to the calorie restriction–induced body weight reduction, rather than to consumption of the MedDiet per se.

We hypothesized that the consumption of the MedDiet under weight-maintaining conditions is associated with a reduction in cognitive restraint and in susceptibility to hunger because of its purported satiating effect [13]. The lack of change in eating behavioral traits with the MedDiet in the absence of weight loss may be explained in part by the specificity of our intervention. Munsch et al. [26] have shown that changes in susceptibility to hunger may still occur 12 months after the end of a lifestyle change program for the treatment of obesity. The isoenergetic MedDiet feeding phases before and after weight loss in the present study were only of a five-week duration, which may have been too short to modify long-acquired behaviors associated with perceptions and feelings of hunger in men with MetS. It is important to note that participants in the present study had relatively low scores of susceptibility to hunger, according to the categorization proposed by Timko [27]. This may also have hindered further change in this eating behavioral trait in response to the MedDiet. Nevertheless, and consistent with data from the present study, Bédard et al. [28] found no changes in hunger and fullness (measured with visual analogue scales) after a four-week isoenergetic feeding of a MedDiet.

The weight loss phase in free-living conditions increased mean cognitive restraint and decreased disinhibition scores, which is consistent with the large body of literature on this topic, demonstrating that weight loss is generally accompanied by increased cognitive restraint and decreased disinhibition [29,30,31,32,33,34]. The increase in cognitive restraint during the weight loss period was mostly related to an increase in participants’ rigid control, contrary to the results of a systematic review where flexible control, but not rigid control, was identified as a mediator of weight control [35]. This change in behavior probably reflects a more pronounced psychological effort from the participants to achieve the assigned weight loss required to remain in the study [30].

Another important dimension that deserves consideration in this study pertains to the change in susceptibility to hunger during the weight loss phase. Previous studies on this topic that have used TFEQ have often observed an unexpected reduction in susceptibility to hunger during weight loss [29,30,31] and our results are consistent with this observation. We have to keep in mind that susceptibility to hunger as measured by TFEQ is a subjective concept that is not always related to appetite sensations measured by visual analog scales [15], which specifically assess hunger, appetite, and satiety as dissociated concepts [36]. We suspect that lower susceptibility to hunger scores may be related to some extent to the concurrent increase in cognitive restraint occurring during weight loss. In fact, increased cognitive restraint is generally associated with an increased control over dietary and behavioral factors associated with body weight regulation. Thus, subjects with high cognitive restraint scores may have also perceived and reported improved control over their sensations and perceptions of hunger because they were successful in achieving significant weight loss. It would be of interest to assess the impact of the MedDiet before and after weight loss on specific measures of appetite and satiety in the future.

Finally, data showed that cognitive restraint scores remained high when participants having achieved >5% weight loss consumed the MedDiet under weight-stabilizing conditions for five weeks. The fact that cognitive restraint did not go back to lower values with the MedDiet after weight loss was unexpected, considering that participants were consuming large servings of highly satiating foods without the concern of gaining weight, knowing that they were being fed to keep their body weight stable. This evidence supports the concept that weight loss has profound effects on eating behavioral traits.

This study has strengths and limitations. The study was undertaken using tightly controlled feeding conditions and results must be interpreted in this context. Assessing the impact of the MedDiet on eating behavioral traits in free-living conditions may yield different results. On the other hand, the unique study design allowed us to assess the impact of the MedDiet on eating behaviors in the presence and absence of body weight loss. The small sample size and the short duration of the intervention may have limited our ability to detect changes in eating behavioral traits, which otherwise may have been more significant. These are secondary analyses of a clinical trial, for which the primary outcome was a change in cardiometabolic risk factors. There was therefore no a priori power calculation done for the eating behavior outcomes.

## 5. Conclusions

In summary, our results suggest that adhering to the MedDiet may have no impact on eating behavioral traits in men with MetS in the absence of significant weight loss. Our data also indicated that a five-week weight stabilization feeding of satiating and low energy foods as part of the MedDiet does not modify the profound, long-term impact of body weight loss on eating behavior traits. As indicated earlier, these results must be interpreted within the controlled, short-term feeding context of the study. Nevertheless, we believe this is the first interventional study documenting the impact of the MedDiet on eating behavioral traits as measured by TFEQ. Eating behavioral traits in men have not been investigated extensively and more studies are needed in this particular group of individuals. Whether longer-term adherence to the MedDiet would trigger changes in eating behavioral traits independent of weight changes has yet to be established.

## Figures and Tables

**Table 1:** Composition of the prescribed North American control diet and MedDiet.

Variable	Control Diet	MedDiet
**MedDiet score** ^a^	20	37
**Key foods** ^b^		
Whole grains products (servings/day)	1.2	5.4
Fruits and Vegetables (servings/day)	6.6	16.1
Legumes (servings/week)	0.6	3.6
Nuts (servings/day)	0.5 ^c^	0.9
Cheese and yogurt (servings/day)	1.8	2.0 ^d^
Fish (servings/week)	1.0	8.8
Poultry (servings/week)	6.8	6.0
Eggs (servings/week)	2.6	2.2
Sweets (servings/week)	13.0	2.0
Red meat (servings/week)	13.1	1.2
Red wine (servings/week)	7.0	17.5
Olive oil (ml/week)	4.5	302.8
**Macronutrient composition/day**		
Proteins (g, (% of kcal))	106 (17)	106 (17)
Lipids (g, (% of kcal))	95 (34)	89 (32)
Carbohydrates (g, (% of kcal))	303 (48)	313 (50)
Total fibers (g)	20	42
Energy density (kcal/g)	1.35	0.93

^a^ Mediterranean (MedDiet) score is on a scale of 0 to 44 (see Methods Section 2.5). ^b^ All numbers are based on 2500 kcal/day. Standardized portion sizes based on Canada’s Food Guide were used: for whole grains products = 125 mL (rice, pasta, bulgur, couscous), one piece of bread or 30 g cereal; Portion size for fruits and vegetable = 125 mL or one fruit/vegetable; Portion size for legume = 175 mL; Portion size for nuts = 30 g; Portion size for fish, poultry and red meat = 75 g; Portion size for egg = 100 g; Portion size for dairy products = 50 g cheese, 175 g yogurt and 250 mL milk; Portion size for red wine = 75 mL. ^c^ Mostly peanut butter on the control diet. ^d^ Low fat cheese and yogurt on the MedDiet.

**Table 2:** Physical characteristics and eating behaviors scores of subjects at baseline (*n* = 19).

Variable	Mean ± SD ^a^	Range
Age (years)	50.8 ± 10.8	24–62
Weight (kg)	100.4 ± 19.7	80.8–155.9
BMI (kg/m^2^)	33.7 ± 5.5	26.9–50.4
Waist circumference (cm)	113.5 ± 12.2	98.7–145.7
MetS (%)	100	-
Cognitive restraint ^b^	7.3 ± 3.4	2–15
Disinhibition ^b^	6.0 ± 2.5	2–11
Susceptibility to hunger ^b^	3.3 ± 3.3	0–12

^a^ SD, standard deviation; BMI, Body Mass Index; MetS, metabolic syndrome; ^b^ Scores as determined by the TFEQ (see methods for detail). Scales vary from 0 to 21 points for cognitive restraint, 0 to 16 points for disinhibition and 0 to 14 points for susceptibility to hunger. Higher scores reflect more pronounced behavioral traits.

**Table 3:** Physical characteristics and eating behavioral traits measured at the end of the different phases of the study.

	Control Diet	MedDiet ^1^ before WL	Free-Living WL Phase	MedDiet after WL	*p* *
**Physical characteristics**					
Body weight (kg) ^†^	99.5 ± 19.7	98.3 ± 19.6	90.4 ± 18.4 ^a,b^	89.4 ± 18.2 ^a,b^	< 0.0001
BMI (kg/m^2^) ^†^	33.4 ± 5.6	33.0 ± 5.5	30.3 ± 5.2 ^a,b^	29.9 ± 5.2 ^a,b^	< 0.0001
Waist circumference (cm) ^†^	110.7 ± 10.2	112.2 ± 12.2	105.2 ± 13.4 ^a,b^	104.3 ± 13.4 ^a,b^	< 0.0001
**Eating behavioral traits** *(range score) ^2^*					
Cognitive restraint (*0*–*21*) ^†^	7.17 ± 4.35	7.23 ± 4.40	10.85 ± 4.23 ^a,b^	10.41 ±	< 0.0001
Flexible restraint (*0*–*7*) ^†^	2.12 ± 1.36	2.44 ± 1.76	3.17 ± 2.12	3.18 ± 1.91	0.05
Rigid restraint (*0*–*7*)	1.79 ± 1.74	1.74 ± 1.56	3.04 ± 1.60 ^a,b^	3.43 ± 2.13 ^a,b^	< 0.0001
Strategic restraint (*0*–*4*) ^†^	0.38 ± 0.92	0.50 ± 0.79	1.06 ± 1.11 ^a^	0.74 ± 0.87	0.008
Attitude to self-regulation (*0*–*5*)	2.93 ± 1.39	2.79 ± 1.32	3.73 ± 0.80 ^a,b^	3.58 ± 1.43 ^a,b^	0.003
Avoidance of fattening foods (*0*–*4*)	1.67 ± 0.84	1.74 ± 1.10	2.29 ± 1.10 ^a^	2.32 ± 1.25 ^a^	0.004
Disinhibition (*0*–*16*)	6.28 ± 2.33	6.07 ± 2.31	5.07 ± 2.18 ^a,b^	5.04 ± 2.30 ^a,b^	0.0003
Habitual (*0*–*5*)	0.47 ± 0.62	0.56 ± 0.62	0.44 ± 0.63	0.47 ± 0.61	0.96
Emotional (*0*–*3*)	1.11 ± 1.29	1.00 ± 1.25	0.59 ± 1.12	0.61 ± 1.09	0.17
Situational (*0*–*5*)	3.00 ± 1.41	2.79 ± 1.23	2.06 ± 1.26 ^a,b^	2.06 ± 1.39 ^a,b^	< 0.0001
Susceptibility to hunger (*0*–*14*) ^†^	3.82 ± 3.67	3.74 ± 3.26	2.45 ± 2.74 ^b^	2.47 ± 2.67 ^b^	0.006
Internal (*0*–*6*) ^†^	1.06 ± 1.57	0.84 ± 1.46	0.50 ± 1.25 ^a^	0.47 ± 1.02	0.03
External (*0*–*6*)	1.79 ± 1.76	1.89 ± 1.70	1.12 ± 1.41 ^b^	1.21 ± 1.36	0.01

^1^ MedDiet, Mediterranean diet; WL, weight loss; BMI, body mass index. Values are presented as means ± standard deviation. ^2^ Higher scores reflect more pronounced behavioral traits. Analyses presented are based on the PROC MIXED procedure in SAS. * *p*-values from the main effect of the diet in the mixed model ^†^ Analysis was performed on log-transformed values. ^a^ Significantly different from control diet, *p* < 0.05. ^b^ Significantly different from MedDiet before WL, *p* < 0.05
